# The Effectivity of a School-Based Early Intervention Targeting Psychological Complaints and Non-Suicidal Self-Injury in Adolescents

**DOI:** 10.3390/jcm13071852

**Published:** 2024-03-23

**Authors:** Imke Baetens, Lisa Van Hove, Zahra Azadfar, Martijn Van Heel, Veerle Soyez

**Affiliations:** Brussels University Consultation Centre (BRUCC), Department of Clinical Psychology, Vrije Universiteit Brussel, 1050 Ixelles, Belgium; lisa.van.hove@vub.be (L.V.H.); zahra.azadfar@vub.be (Z.A.); martijn.van.heel@vub.be (M.V.H.); veerle.soyez@vub.be (V.S.)

**Keywords:** school-based, universal, prevention, psychological symptoms, non-suicidal self-injury, suicidality

## Abstract

**Background**: Recent research suggests a concerning trend of non-suicidal self-injury (NSSI) and suicidal behaviors emerging at younger ages (as early as age 12). Early onset of NSSI is linked to more severe outcomes. While universal school-based prevention programs have shown promise in addressing suicidal behaviors, there is limited research on their effectiveness in preventing NSSI onset among adolescents. This study aims to evaluate the efficacy of a universal prevention program in schools for NSSI and mental complaints while enhancing resilience and mental health in 11–14-year-old adolescents. **Methods**: In total, 329 Flemish secondary school students (55.6% female), aged 11 to 14 years, participated in a 4 h classroom universal prevention, with a focus on emotion regulation, mental health, and specific strategies to prevent NSSI and reduce stigma. For both the intervention and control group (*N* = 124), a pre-, post-, and one-month follow-up questionnaire was administered, containing reliable and valid measures for NSSI and suicidality, emotion regulation, help-seeking behaviors, well-being, and psychological distress. **Results**: The prevention program effectively reduced NSSI and psychological distress, particularly for adolescents with a history of NSSI. **Conclusions**: These findings support previous research on the effectiveness of school-based programs in reducing mental complaints and suggest promising outcomes for NSSI prevention.

## 1. Introduction

Worldwide, one in seven adolescents meets diagnostic criteria for mental health disorders, with depression, anxiety disorders, and behavioral problems being the most common [1]. About 55% of adolescents experience mild to severe psychological symptoms at least once a week in Belgium [2]. Furthermore, some researchers suggest that psychological symptoms, such as depressive and anxiety symptoms, have increased in adolescents compared to pre-pandemic estimates [3,4,5]. This is worrying, as adolescent mental health symptoms have been linked to adverse long-term outcomes, such as failure to complete high school [6], criminality [7], and unemployment [8] in adulthood.

Additionally, increasing rates of suicidal thoughts and behaviors in adolescents have been reported in numerous countries in the aftermath of the COVID-19 pandemic [9]. Data from a Belgian sample reported suicidal ideation in the past few weeks in 15% of adolescents (aged 15–25) [10]. Numbers in Flanders (the Flemish-speaking part of Belgium) are comparable, with a lifetime prevalence for suicidal ideation of 22.3% for 11- to 18-year-olds [11]. Although the last years brought a decreasing trend in suicide rates, Flanders has seen an increase in suicide in the 15–29-year-old female group [12]. According to a recent study from a Belgian sample, less than 50% of individuals who suffer suicidal ideation sought the help of a health professional in the past 12 months [10]. There is a trend towards earlier onset of suicidal thoughts and behaviors [13].

Similarly, a trend in earlier ages of onset for NSSI has been observed in the past years [13,14]. Non-suicidal self-injury (NSSI), or the intentional and direct damage to one’s own bodily tissue without suicidal intent and without cultural aspects [15], is a pressing concern in many educational settings around the world. Lifetime prevalence rates of NSSI in nonclinical adolescent samples range between 17 and 38% in several meta-analyses [16,17,18]. A recent meta-analysis [19] identified a trend towards more severe NSSI in the past decade. Furthermore, several studies underscore that adolescents start engaging at an increasingly younger age [13]. Adolescents who began self-injuring at or before age 12 reported significantly more lifetime acts of NSSI, greater versatility of the method, and medically more severe NSSI than those who started NSSI around age 17 and older [20]. Research has indicated that the expectation of future engagement in NSSI was predictive of NSSI recurrence [21], subsequently influencing potential lethality and the variety of NSSI methods used [22]. Furthermore, NSSI has been identified as a predictor of mental health disorders and suicidality in particular, especially when the behavior is engaged repetitively [23,24]. On the contrary, mental health problems are important risk factors for the development and maintenance of NSSI [25,26,27]. A possible explanation for the association between mental health problems and NSSI behavior is the lack of adequate emotion regulation strategies [28]. For example, te Brinke et al. [29] found that adolescents who reported both externalizing (e.g., rule-breaking and aggression) and internalizing problems (e.g., anxiety and depression) adopted a maladaptive regulation style, which in turn is a maintaining factor for NSSI [26]. Furthermore, NSSI itself can be seen as a maladaptive regulation style [30,31]. By participating in NSSI, adolescents can find an alternative way to cope with distressing situations, alter their social environment, gain relief from negative emotions or thoughts, or change self-cognitions [32].

Although mental health problems and NSSI are common among adolescents, only 17.5% of 16- to 18-year-olds seek professional help [33]. This may be due to adolescents encountering stigmatic barriers when considering seeking help, such as negative beliefs about professional mental health care [34], negative responses to disclosure [35,36], and lack of knowledge about where to seek help [37]. Furthermore, more severe mental health problems, including NSSI and suicidality, lead to a greater delay in seeking help [38]. For example, according to a recent study from a Belgian sample, less than 50% of individuals who suffer from suicidal ideation sought the help of a health professional in the past 12 months [10]. Some factors identified to date that promote help-seeking behavior [39] are mental health literacy [40,41] and appropriate responses to disclosure of mental health issues [42,43]. The prevalence rates, adverse long-term outcomes of mental health problems in adolescents, and low rates of help-seeking behavior call for effective preventative and early interventions to decrease mental health complaints and NSSI in adolescents.

The school setting may be an opportune context to implement such preventative and early mental health interventions, as they can take place where adolescents spend a large portion of their time [44] and reach a larger group of children who are potentially at risk for developing mental health problems or participating in NSSI [45]. It also allows us to educate peers on mental health (mental health promotion (MHP)) and on responses to mental health issues, which in turn leads to less stigma and more help-seeking behavior [46,47,48,49].

Several meta-reviews over the past decades [50,51] found the benefits of classroom-based preventative and mental health psychological interventions. One of the major benefits is that they target the entire student population at a relatively low cost [52]. Studies have also shown the promising effectiveness of universal school-based programs in promoting mental health and strengthening resilience while mitigating psychological problems among students. For adolescent student groups specifically, universal school-based prevention programs have been extensively researched to evaluate their effectiveness in improving academic success [44,53], addressing positive mental health outcomes, including building resilience, coping behavior, and subjective well-being [44,54,55,56], or improving emotion regulation skills [57], as well as addressing a range of mental health issues such as substance misuse [58], suicidality [59], and other mental health concerns [60,61,62]. For example, the systematic review and meta-analysis of Tejada-Gallardo et al. [63] found evidence for the efficacy of school-based multicomponent positive psychology interventions in improving mental health in the short and long-term with small effect sizes ranging from g = 0.2 to g = 0.3 for subjective well-being, psychological well-being and depressive symptoms. 

Notwithstanding the bulk of evidence concerning a variety of mental health outcomes, there are only two studies that focused on the effectivity of universal school-based programs with regard to NSSI. One such intervention is the so-called Peer Education Programme (NSSI-PEP) [64], which targets four key risk factors for NSSI, namely pubertal change, body image, self-esteem, and emotion regulation, found positive changes in emotion regulation skills, self-esteem, and fear. Another intervention is the “HappylesPLUS” by Baetens et al. [65]. Baetens et al. [65] investigated the outcomes of an in-classroom universal mental health program “Happyles” [62] and compared it with the outcomes of an enriched program, with a one-hour NSSI-focused psychoeducation module (“KRAS”). This module addresses the social contagion mechanisms of NSSI, both among peers and online and aims to reduce the stigma surrounding NSSI, with the goal of encouraging more proactive behavior in seeking and offering help. This is accomplished by providing psychoeducation about NSSI (e.g., functions of NSSI; open wounds caused by NSSI may incite others) and discussing effective ways to talk about NSSI and what to do if a student suspects or knows someone is engaging in NSSI.

Both groups reported a reduced tendency for future NSSI and improved emotional awareness six weeks after the program completion, compared with the pre-test results. Qualitative analysis of the data suggested that “HappylesPlus” may provide direct benefits for students who actively engage in NSSI, such as a greater willingness to seek help for this behavior [65].

Another shortcoming is that most existing prevention programs either focus on positive mental health/positive psychology interventions [63] or on interventions strengthening mental health literacy [66]. However, when aiming to target the whole class population, it is best to build on a dual-continuum model of mental health [67]. This model has as its premise that mental illness and positive mental health predict and explain different outcomes (which indicates that these are two distinct constructs), while simultaneously, these constructs share some degree of overlap. When measuring functioning on both constructs, four distinct “at-risk” subgroups can be distinguished. This brings significant potential for intervention development [68]. More specifically, a so-called multi-tiered intervention, with three tiers (that are commonly called universal, selective, and indicated), can be adopted [69]. However, more research on the effectivity of multitiered school-based interventions that focus on both the promotion of mental well-being and the prevention of mental health problems, and NSSI specifically, is needed. 

The current study therefore aims to further contribute to the largely unexplored question of the effectiveness of multi-tiered school-based early interventions that both target universal resilience building and mental health literacy. Apart from the earlier work of Baetens et al. [65] and Cipriano et al. [64], there is an important gap in research to examine the effectiveness of school-based early intervention programs to prevent NSSI in schools. Since the initial pilot study by Baetens et al. [65], the Flemish Institute for Healthy Living, a center of expertise for health promotion, has launched a guide model for mental health promotion (“The Happiness Triangle”) and a related intervention for educational settings (“Happiness in the classroom”). This intervention contains a psychoeducational package comparable to the “Happyles” program [62] evaluated in the pilot study. However, “Happiness in the classroom” as a whole, nor its separate elements, has not yet been quantitatively examined for effectiveness.

Therefore, the main purpose of this study is to investigate the effectiveness of a multi-tiered school intervention combining the Happiness Triangle psychoeducational package with the KRAS psychoeducational module for NSSI and subsequent tailored advice on an individual level [65]. More specifically, the current study investigated whether the level of psychological symptoms (i.e., internalizing and externalizing symptoms) in young adolescents (11–14 years) is significantly reduced and the level of mental well-being significantly improved in students who followed the school-based early intervention program compared to the control group. Additionally, we examine whether the program has an effect on reducing the likelihood of engaging in NSSI and increases both help-seeking behavior and emotion regulation strategies compared to the control group. 

In line with recent studies involving Flemish adolescents in similar age ranges, we expect 50% to report psychological symptoms [2] and 7% to report NSSI in the past year [70]. We expect an overall positive well-being for the majority of the group and no more than 20% to report low well-being [2]. Furthermore, the majority of Flemish adolescents in the first grade are expected to have primarily adaptive emotion regulation strategies, with only a minority reporting difficulties in emotion regulation. Flemish adolescents are often not very interested in help-seeking behaviors for mental health problems. 

Furthermore, based on previous studies on universal prevention programs [59,65], we expect that a universal prevention program (with a specific NSSI-focused module KRAS) has a positive effect on rates of internalizing and externalizing symptoms and NSSI and decreases in emotion regulation problems, and finally has a positive impact on help-seeking attitudes.

## 2. Materials and Methods

### 2.1. Procedure

First, ethical approval was obtained from the Brussels UZ Medical Ethics Committee (Brussels, Belgium) (BUN: 1432022000257). From January to May 2023, a universal psychoeducational package called Happiness Triangle, as developed by the Flemish Institute for Healthy Living (Brussels, Belgium), was installed in the first and second grades of six secondary schools in Flanders by the prevention team of Vrij CLB Brabant Oost (Aarschot, Belgium). The program combines a class-based universal prevention package focusing on resilience (including 3 classroom hours on well-being, resilience, coping, and help seeking), a psychoeducational module on NSSI (KRAS module) [65], and tailored advice on an individual level. The program and the brief counseling (15 min per student) were delivered by a team of 3 local school counselors (of vCLB Brabant Oost, Brussels, Belgium). 

Before lessons, students and their parents received an information sheet and informed consent form from their teacher. If both parents and students consented to participate, the students were asked to individually complete a pseudo-anonymous, online pre-, post-, and follow-up self-report questionnaire via a secure web survey platform (i.e., Qualtrics XM). The questionnaire enquired about demographics, NSSI and suicidality, emotion regulation, internalizing and externalizing symptoms, help-seeking behavior, and mental health stigma. The pre-questionnaire was completed before the first lesson, the post-questionnaire was completed immediately after the fourth lesson, and the follow-up questionnaire was completed one month after the post-questionnaire. All participating students were randomly assigned an ID code, which they had to fill in at the beginning of each questionnaire. This allowed the researchers to link responses across timepoints without compromising student anonymity. In addition to the intervention group, a group of students who had not participated in the prevention classes were also asked to fill in the questionnaire at three timepoints after receiving informed consent from both the students and their parents. These students, who were matched by age, region, and educational level, served as the control group. 

Each student was also given an open invitation to a brief 15 min individual counseling session at school with a member of the school counseling team. During this session, students had the opportunity to express their questions, and those who exhibited an elevated risk profile (e.g., increased psychological complaints and decreased mental well-being) were referred to professional support. Students with immediate risk (e.g., acute suicide risk) were referred to a crisis center and closely followed up by the school counselor center. Finally, incentives (i.e., movie tickets) were distributed to 50 randomly selected students, and a brief overview of the study results was sent by email to students who requested it.

### 2.2. Participants 

In total, 329 students from the early intervention group participated in the pre-measurement, 242 students in the post-measurement (26.4% dropouts), and finally 166 students in the follow-up measurement (50.8% dropouts). Overall, for 62 cases, the questionnaires could be linked to each other via a pseudo-anonymized code (53.2%, female, Mean age = 12.66, SD = 0.673, min. = 11, max. = 14) for the three timepoints (25.62%//18.9% of the total)). 

Regarding the control group, 185 students completed the questionnaire in T1, 184 in T2 (0.54% dropouts), and 183 in T3 (1.08% dropouts), of which 101 cases could be linked for the 3 timepoints (54.89% of 185) (55%, female, M age = 12.16, SD = 0.518, min. = 11, max. = 14). 

To obtain an equal sample size in both the early intervention and control groups, we randomly selected 62 cases from 101 participants in the control group. To ensure that there are no significant differences in the baseline characteristics of the participants who dropped out and the students who participated in the three assessments, we analyzed the variations between groups. Results indicated no significant differences in terms of mean age and gender between them (*p* > 0.05) (see Table 1 below).

### 2.3. Measures 

Non-suicidal self-injury (NSSI). Participants received the Brief Non-Suicidal Self-Injury Assessment Tool (BNSSI-at) [71]. Items regarding NSSI methods, functions, recency, frequency, age of onset, cessation, and probability of future engagement were enquired. The test–retest reliability and validity of the NSSI-AT is adequate [72].

Emotion regulation. How students regulate their emotions was measured with the Difficulties in Emotion Regulation Scale (DERS-36) [73]. The DERS-36 contains 36 items on a 5-point Likert scale (5 = almost never to 1 = almost always). In a sample of adolescents specifically, the subscales showed good to excellent internal consistency [74]. In the current study, the internal consistency of the total score was excellent, with Cronbach’s alpha values of 0.87 at T1, 0.91 at T2, and 0.92 at T3. The internal consistency of the subscales was also in the acceptable to good range: Lack of Emotional Clarity (α = 0.76 at T1, 0.80 at T2, and 0.85 at T3), Difficulties Engaging in Goal-Directed Behavior Goals (α = 0.76 at T1, 0.84 at T2, and 0.86 at T3), Impulse Control Difficulties (α = 0.82 at T1, 0.83 at T2, and 0.82 at T3), Limited Access to Effective Emotion Regulation Strategies (α = 0.76 at T1, 0.84 at T2, and 0.79 at T3), and Non-Acceptance of Emotional Responses (α = 0.69 at T1, 0.78 at T2, and 0.64 at T3).

Mental well-being. The Warwick–Edinburgh Mental Well-being Scales (WEMWS) [75] were administered to gain insight into participants’ general mental well-being. It consists of 14 items with a 5-point Likert scale (0 = none of the time to 4 = all of the time). The sum of the item scores is calculated to obtain a total score. The internal consistency of the WEMWS in the current study was good (α = 0.90 at T1, 0.92 at T2, and 0.94 at T3). 

Psychological symptoms. To track internalizing and externalizing symptoms, the brief self-report version of the Youth Outcome Questionnaire (Y-OQ-SR 30.2) [76,77] was used. The Y-OQ-SR 30.2 has 30 items on a 5-point Likert-type scale (0 = almost never or never to 4 = almost always or always), a score range of 0 to 120, and can be divided into six subscales: somatic, social isolation, aggression, conduct problems, hyperactivity/distractibility, and depression/anxiety. The higher the total score, the greater the distress experienced by the participant. For both the total score and the subscales, internal consistency and test–retest reliability were found to be adequate in a community youth sample [77]. In the current study, Cronbach’s alpha for the total score was excellent (α = 0.91 at T1, 0.93 at T2, and T3). The internal consistency of the subscales somatic problems (α = 0.70 at T1, 0.75 at T2, and 0.77 at T3), conduct problems (α = 0.79 at T1, 0.80 at T2 and 0.78 at T3), and depression/anxiety (α = 0.81 at T1, 0.85 at T2, and 0.82 at T3) was good. The internal consistency of social isolation (α = 0.68 at T1, 0.70 at T2 and 0.68 at T3), aggression (α = 0.71 at T1, 0.72 at T2, and 0.63 at T3), and hyperactivity/distractibility (α = 0.64 at T1, 0.68 at T2, and 0.69 at T3) was acceptable. 

Depressive symptoms. The Centre for Epidemiologic Studies Depression Scale (CES-D) [78] was administered to identify the presence and extent of depressive feelings or symptoms. The questionnaire consists of 20 items, which are answered on a 4-point Likert scale (0 = seldom or never (less than one day) to 3 = almost always or always (5–7 days)), and includes the following components: depressed mood, feelings of guilt and inferiority, feelings of helplessness and despair, loss of appetite, sleep disturbances and psychomotor delay. Cronbach’s alpha for the CES-D in the current study was good (α = 0.92 at T1, 0.84 at T2, and 0.85 at T3).

Help-seeking behavior. The help-seeking behavior of students was assessed using the Short Form Attitudes Towards Seeking Professional Psychological Help Scale (ATSPPHS-SF) [79]. The ATSPPHS-SF is a unidimensional instrument with 10 items (e.g., “If I believed I was having a mental breakdown, my first inclination would be to get professional attention”) that are answered on a 5-point Likert scale (1 = completely disagree to 5 = agree completely) [79]. Higher scores indicate more positive attitudes towards seeking professional help, which has been associated with greater willingness to engage in future help-seeking behavior and less stigma related to treatment. In several studies, adequate internal consistency and test–retest reliability have been found [80]. Although these studies were conducted on college student samples, researchers concluded that no items were included that would be inapplicable or inappropriate in an adolescent sample [81]. In the present study, Cronbach’s alpha for the total score was 0.64 at T1, 0.76 at T2, and 0.80 at T3.

### 2.4. Data Analysis

Descriptive statistics for the final sample were reported for the study variables, with means and standard deviations reported for the continuous variables (i.e., difficulties in emotion regulation, depressive symptoms, mental well-being, internalizing and externalizing problems, and help-seeking behavior) and sample distribution for the ordinal and nominal variables (i.e., the prevalence of NSSI and suicide attempts). Furthermore, the differences between the intervention group and the control group were assessed at T1. Independent samples t-tests were performed to test for differences in the continuous variables, whereas chi-square tests were used to compare ordinal and nominal variables.

To evaluate the changes between pre-, post-, and follow-up measures, repeated measure ANOVAs were used for the continuous data. In these ANOVAs, a time factor was included, which entails running an omnibus test of differences across timepoints. Furthermore, an interaction term between time and group was included in order to assess whether the differences across timepoints differed between the experimental and control groups. To obtain a more detailed insight, post hoc analyses were conducted. First, to assess within-group differences across timepoints in detail, separate ANOVAs in each group were conducted, and contrasts were applied via the Bonferroni post hoc test to investigate pairwise differences between timepoints. Furthermore, the interaction effect between time and group was assessed in more detail by testing the differences scores between timepoints (i.e., pre- vs. post; post- vs. follow-up measures) between groups (i.e., intervention vs. control group) using ANOVAs. Before performing repeated measures ANOVAs, the assumption of normality of the data was tested using the Kolmogorov–Smirnov test, which confirmed the normal distribution of most variables in both the intervention and control groups (*p* > 0.05). However, in both groups, depressive symptoms at all three timepoints, internalizing and externalizing problems at T2 and T3, and help-seeking behavior at T3, as well as the data for the internalizing/externalizing problems at T1 in the control group, were not normally distributed and were normalized using the fractional rank method. Additionally, the assumption of sphericity of the test statistics was tested using the Mauchly test of sphericity, and the results were not significant (*p* > 0.05) for most variables, implying that the variances of the differences between all combinations of related groups (levels) are equal. In the case of significant differences between the variances, the Greenhouse-Geisser was applied. 

Ordinal and nominal variables (i.e., suicide and NSSI prevalence) were analyzed using the nonparametric Mann–Whitney U test and the Wilcoxon signed-rank test. Partial Omega Squared (ω2p) was calculated to determine effect sizes and interpreted as 0.01 = small effects, 0.06 = moderate effects, and 0.14 = large effects. Ω2p is a less biased version of partial eta-squared (η2p) for ANOVAs [82]. All analyses were performed using SPSS (version 29), and statistical significance was determined with an alpha level of 0.05.

## 3. Results

### 3.1. Descriptive Statistics

Regarding difficulties in emotion regulation, depressive symptoms, mental well-being, internalizing and externalizing problems, and help-seeking behavior, the descriptive statistics at T1 are presented in Table 2. Comparing the intervention and control group at T1, a significantly higher score was observed in the intervention group for internalizing and externalizing problems, whereas the control group scored significantly higher on mental well-being and help-seeking behavior (see Table 2).

Based on the data from the baseline, the lifetime prevalence of suicide attempts and NSSI in participants was 4.8% (*n* = 25) and 22% (*n* = 115), respectively. The total sample showed a mean score of 36.44 (SD = 11.36) for difficulties in emotion regulation; 53.2% of the sample were in the clinical range. In terms of internalizing and externalizing problems (M = 27.48, SD = 17.21), 15.1% of the total sample was identified as subclinical, and 40.3% were in the clinical range. Additionally, based on the data from the CES-D measure (M = 15.44, SD = 11.04), 10.9% were in the subclinical, and 26.8% were in the clinical range for depressive symptoms. In terms of help-seeking behavior (M = 30.85, SD = 5.72), 67.9% were below the cut score of positive attitudes for seeking professional help. Among participants with a history of NSSI, 9.2% reported committing NSSI for more than 5 days in the past year, 21.4% reported having engaged in NSSI at least once in the past year, and 13.4% reported having engaged in NSSI at least once in the past month. There were no significant differences in the prevalence of lifetime NSSI between intervention and control groups (χ^2^ (1) = 0.954, *p* = 0.329). Furthermore, the results of the Mann–Whitney U test did not show significant differences between the participants in the intervention group and the control group in the number of days they participated in NSSI during the last 4 weeks (Z = −0.74, *p* = 0.459).

### 3.2. Repeated Measures ANOVA

**Figure 1:**
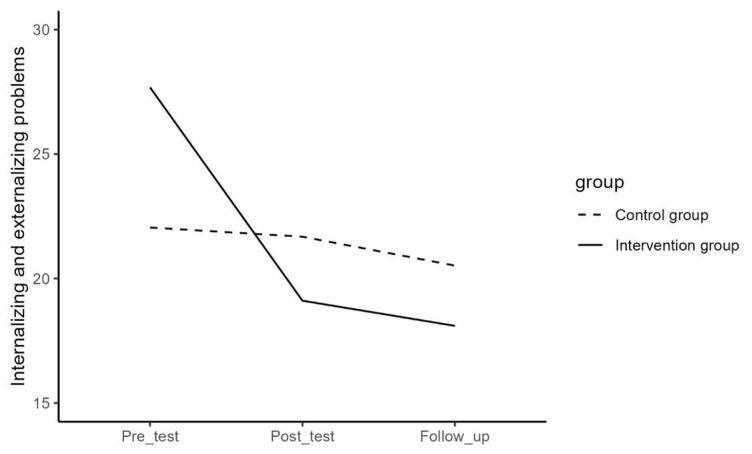
The effect of time on internalizing and externalizing problems in the intervention and control group.

**Figure 2:**
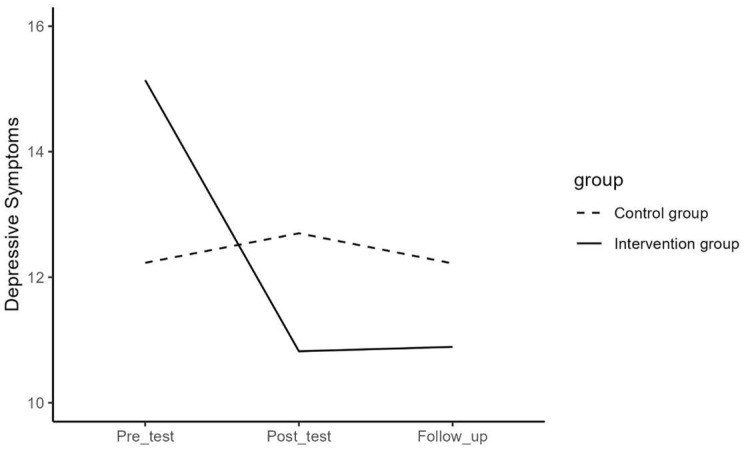
The effect of time on depressive symptoms in the intervention and control group.

**Figure 3:**
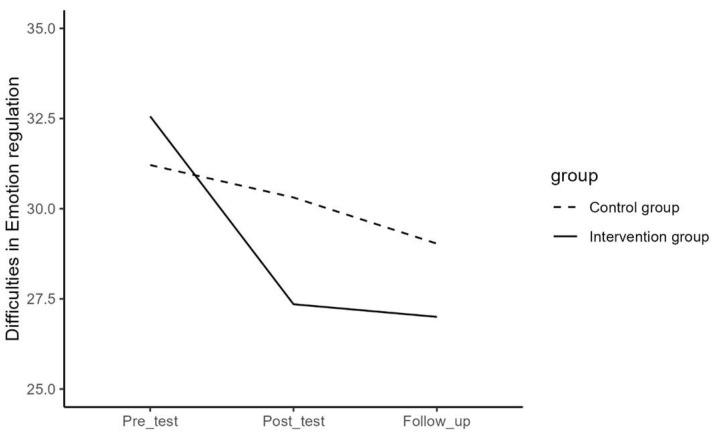
The effect of time on difficulties in emotion regulation in the intervention and control group.

**Figure 4:**
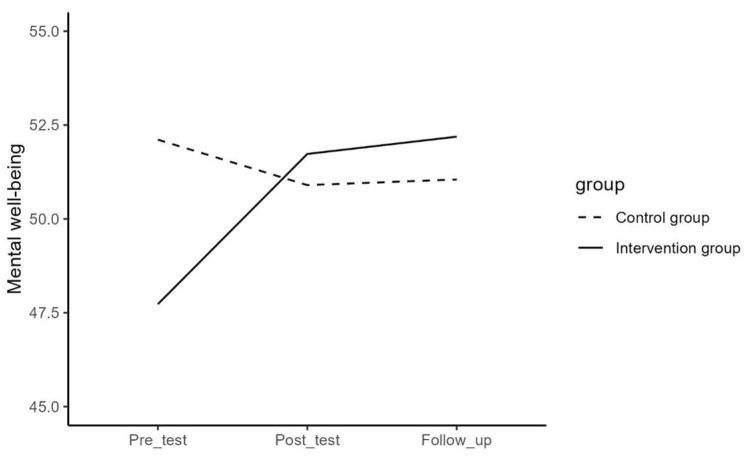
The effect of time on mental well-being in the intervention and control group.

**Figure 5:**
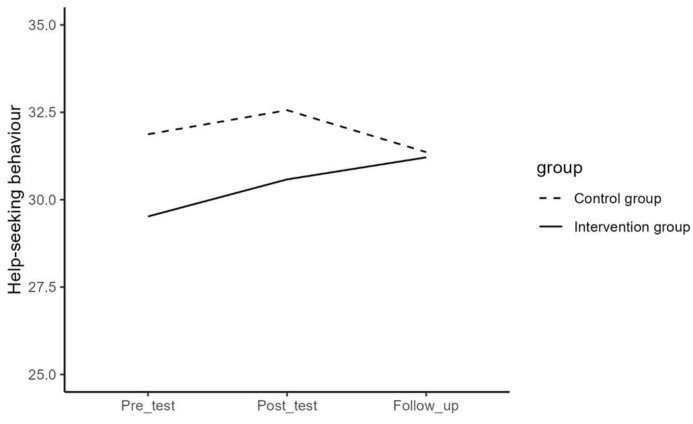
The effect of time on help-seeking behavior in the intervention and control group.

Regarding difficulties in emotion regulation, depressive symptoms, mental well-being, internalizing and externalizing problems, and help-seeking behavior, the results of the repeated measures ANOVA for the effect of time and the interaction effect of time and group are presented in Table 3 and visualized in Figure 1, Figure 2, Figure 3, Figure 4 and Figure 5. The results showed that the effects or changes in the mean scores of difficulties in emotion regulation, depressive symptoms, and internalizing and externalizing problems over time were significant at an alpha level of 0.001 and for mental well-being at an alpha level of 0.005. Changes in help-seeking behavior were not significant across time. Furthermore, a significant interaction term indicated that the change in mean scores of these variables differed significantly between the intervention and control groups (*p*< 0.05).

To examine the effect of time in each group, a series of repeated measures ANOVA and the Bonferroni post hoc test were performed separately for the intervention and control groups. The average values per timepoint in the separate groups are presented in Table 4. 

In the intervention group, the results showed significant changes in the mean scores of difficulties in emotion regulation (F = 25.00, *p* < 0.05), depressive symptoms (F = 21.17, *p* < 0.05), mental well-being (F = 15.76, *p* < 0.05), internalizing and externalizing problems (F = 31.34, *p* < 0.05), and help-seeking behavior (F = 3.97, *p* < 0.02) over time. Pairwise contrast analyses revealed that there was a significant decrease between pre- and post-measure, but not between post- and follow-up measures for difficulties in emotion regulation, depressive symptoms, and internalizing and externalizing problems. For mental well-being, there was a significant increase between pre- and post-measures, but not between post- and follow-up measures, and for help-seeking behavior, there was a significant increase between pre- and follow-up measures, but not between pre- and post-measures. The results of the Bonferroni post hoc test for pairwise comparisons of the T1, T2, and T3 measurements in the intervention group are presented in Table 5. 

In the control group, the results showed significant changes in the mean scores of difficulties in emotion regulation (F = 3.75, *p* < 0.05) over time. There were, however, no significant changes in mean score in depressive symptoms (F = 0.34, *p* = 0.710), mental well-being (F = 1.85, *p* = 0.160), internalizing and externalizing problems (F = 1.70, *p* = 0.190), and help-seeking behavior (F = 1.39, *p* = 0.290). Pairwise contrast tests revealed that the mean scores of difficulties in emotion regulation for the participants in the control group have decreased significantly from pre- to follow-up (*p* < 0.05) but not from post- to follow-up measures (See Table 6).

In order to examine the interaction effect between time and group in detail, contrast analyses tested whether the change between pre- and post-, post- and follow-up measurements, and between pre- and follow-up significantly differed between the intervention and the control group (See Table 7). Results showed that the intervention group had a significantly stronger decrease in difficulties in emotion regulation, depressive symptoms, and internalizing and externalizing problems than the control group between pre- and post- (*p* < 0.001) and between pre- and follow-up measurements (*p* < 0.01). There were no significant differences between post- and follow-up measurements (*p* > 0.05). Furthermore, it was observed that the intervention group had a significantly stronger increase in mental well-being than the control group between pre- and post- and between pre- and follow-up measurements (*p* < 0.001) but not between post- and follow-up (*p* > 0.05) and that the intervention group showed a significantly stronger increase in help-seeking behavior than the control group between pre- and follow-up and between post- and follow-up measurements (*p* < 0.05), but not between pre- and post- (*p* > 0.05). 

### 3.3. Wilcoxon Signed-Rank Test 

The results of the Wilcoxon Signed-Rank Test revealed that the participants in the intervention group showed a significant decrease in the number of days they participated in NSSI in the last 4 weeks from T1 to T3 (Z = −2.39, *p* < 0.05) and also from T2 to T3 (Z = −2.04, *p* < 0.05). However, participants in the control group reported that the number of days they had committed NSSI in the previous 4 weeks did not change significantly from T1 to T2 (Z = −1.73, *p* = 0.084), T1 to T3 (Z = −0.59, *p* = 0.558), and from T2 to T3 (Z = −0.88, *p* = 0.380). 

In terms of perceived probability of future involvement in NSSI, which was only asked of participants with a history of NSSI, the intervention group reported a lower probability of participating in NSSI acts or higher resistance against NSSI from T1 to T3 measurements (Z = −2.51, *p* < 0.05). However, participants with a history of NSSI in the control group did not show significant changes in the perceived probability of future participation in NSSI from T1 to T2 (Z = −0.68, *p* = 0.498) and T1 to T3 (Z = −0.77, *p* = 0.441)

## 4. Discussion

The current study focuses on assessing the effectiveness of a multi-tiered early school-based intervention for strengthening mental well-being, emotion regulation skills, and help-seeking behaviors in adolescents on the one hand and preventing NSSI and mental health problems in general on the other. Given the increase in psychological symptoms and maladaptive behaviors (such as NSSI and suicidality) in young adolescents, this study addresses an important societal concern. It is the first study to examine the effectiveness of the universal school-based psychoeducational package developed by Vlaams Institution Gezond Leven, which is freely available for all Flemish schools. Furthermore, it is one of the first studies to explicitly examine the effectiveness of a multi-tiered school program in the prevention of NSSI. 

In line with our hypothesis and post-COVID-19 literature, the prevalence of psychological symptoms and maladaptive behaviors such as NSSI and SSI [11,83,84], depression, and internalizing and externalizing problems [3,85,86,87] remain high, even in 2023. In the current sample, 40.3% of adolescents meet the clinical range for internalizing and externalizing symptoms. In total, 55.4% report psychological distress, approximately 50% exhibit depressive symptoms, 22% engage in NSSI, and 4.8% report a suicide attempt. The prevalence rates of NSSI and suicidality are higher than expected among these young adolescents (11–14 years old). In addition, the majority of adolescents (around 70%) reported negative attitudes towards help-seeking. Flemish adolescents are, in fact, not keen on helping to solve mental health problems.

The Happiness Triangle in combination with the KRAS module showed promising results, where the intervention group after 4 classroom hours of early intervention showed a significant decrease in internalizing and externalizing symptoms, and more specifically depressive symptoms and frequency of NSSI. In addition, the adolescents in the intervention group showed significant improvements in emotion regulation skills and help-seeking behaviors compared to participants in the control group. The findings are consistent with previous research, supporting the mental health benefits of school-based programs in adolescents [44,54,55,56,57,58,59,60,61,62,63,88].

Notwithstanding the strengths of this study (e.g., multi-component program, naturalistic design) and robust findings in line with previous studies, there are several limitations that we would like to acknowledge as well. First, the response rate of 50% was rather low, which is often the case in naturalistic intervention studies. Although we are not aware of a systematic bias, low response rates can threaten the generalizability of the results to all students. Furthermore, it is essential to note that less than 30% of the total sample, could be linked via the pseudo-anonymized coding system. Despite this limitation, comparisons between on group levels yielded similar results. Secondly, the lack of blinding in the allocation of participants to the intervention and control groups introduces the potential for bias, particularly considering that schools had autonomy in enrolling classes for the intervention study. While the prevalence of psychological symptoms and maladaptive coping behaviors persisted at levels consistent with those observed during the COVID-19 pandemic, it is imperative for future research to validate these findings and consider whether participating schools may be disproportionately affected by such issues and were therefore motivated to participate in this study. Furthermore, as this study evaluates the implementation of the program to pre-adolescents ages 11–14, its generalizability to other age groups or students in vocational tracks is limited. Future research endeavors should prioritize the development of more interactive prevention programs that cater to diverse demographics, including refugees and vocational track students. Considering the positive results observed in the current program, key components worth considering for inclusion involve providing psychoeducation on mental health and resilience (focusing on self-acceptance, connection with others, well-being, and coping with difficult events/emotions) while also promoting help-seeking behaviors. Key elements in the KRAS module are decreasing stigma (via class discussions following a documentary of 20 min), providing information on how to deal with NSSI pictures on social media, and how to respond to friends who self-injury. It is important to note that the present study exclusively assessed the overall effectivity of the program. Therefore, to better understand the specific mechanisms at work, future research should investigate the effectivity of specific program elements. Finally, the collaboration with a local school counselor center facilitated program delivery and data administration; however, the absence of adherence to the program records poses a notable limitation, despite the positive outcomes observed.

Notwithstanding these limitations, our study adds to our knowledge of the potential effectivity of school-based early interventions and informs academia on the potential benefits of a multicomponent approach to the prevention of NSSI behaviors in early adolescence. With the high prevalence of psychological symptoms and maladaptive behaviors among school-aged populations, multi-component universal school prevention can counter the current pressure sensed in all inpatient and outpatient child and adolescent psychiatry services, which manifest in lengthy waiting lists and overwhelming burdens on existing resources. By demonstrating the efficacy of school-based prevention programs in improving mental well-being and reducing the incidence of NSSI, our findings offer a proactive approach to alleviating this burden. Implementing such interventions has the potential to not only mitigate the strain on mental health services but also to promote early intervention and support for students at risk, thereby fostering healthier outcomes and reducing the demand for specialized psychiatric care in the future. This relevance underscores the critical importance of integrating preventive measures within educational settings to address the growing mental health needs of young individuals.

## 5. Conclusions

In conclusion, despite the identified limitations, the findings of our study present promising implications for the efficacy of universal school-based programs aimed at addressing NSSI and fostering mental well-being among students. Remarkably, even with a relatively brief intervention duration of just four hours, our results demonstrate tangible improvements in mental health outcomes. These outcomes underscore the potential effectiveness of such interventions in promoting resilience and mitigating psychological distress within school settings. While further research is warranted to address the identified limitations and ascertain the long-term sustainability and generalizability of these findings, our study offers valuable insights into the feasibility and impact of universal school-based programs in fostering a supportive environment conducive to students’ mental well-being and prevention of NSSI behaviors.

## Figures and Tables

**Table 1:** Analysis of the demographic disparities between dropped out and staying participants.

	Intervention Group				Control Group			
	Dropped Out %	Stayed in Study	Value	*p*	Dropped Out %	Stayed in Study	Value	*p*
Gender								
Male	42.9%	48.4%	X2 (1.01)	0.604	55.6	45.2%	X2 (1.82)	0.177
Female	56.3%	51.6%			44.4	54.8%		
Other	0.7%	0.0%			0.0%	0%		
Age (mean)	13.21	13.16	t (−0.72)	0.474	12.31	12.16	t (−1.9)	0.059

**Table 2:** Descriptive statistics of the continuous variables and comparison between control and intervention group at pre-test.

	Total		Control		Intervention		Difference Test	
	M	SD	M	SD	M	SD	*t* (df)	*p*
Difficulties in Emotion regulation	31.89	10.07	31.21	10.92	32.56	9.18	0.748 (122)	0.456
Depressive symptoms	13.45	9.41	11.92	0.987	14.98	8.75	1.830 (122)	0.070
Mental well-being	49.92	8.61	52.11	9.01	47.73	7.66	−2.922 (122)	0.004
Internalizing and externalizing problems	24.87	14.86	22.06	14.96	27.67	14.33	2.136 (122)	0.035
Help-seeking behavior	30.69	5.66	31.87	4.69	29.52	6.31	−2.358 (122)	0.020

**Table 3:** Repeated measures ANOVA: the effect of time and the interaction effect of time and group on continuous variables.

	Source	SS	df	MS	F	*p*	ω2p	Power
Difficulties in Emotion Regulation	Time	1032.909	2	516.454	23.55	<0.001		1
	Time × Group	318.919	2	159.46	7.27	<0.001	0.09	0.934
	Error (Time)	5350.839	244	21.93				
Depressive symptoms	Time	342.467	2	171.233	10.89	<0.001		0.990
	Time × Group	425.386	2	212.693	13.53	<0.001	0.17	0.998
	Error (Time)	3836.978	244	15.725				
Mental Well-being	Time	204.016	2	102.008	5.33	0.005		0.836
	Time × Group	597.79	2	298.895	15.61	<0.001	0.19	0.999
	Error (Time)	4672.86	244	19.15				
Internalizing and Externalizing Problems	Time	2150.563	2	1075.28	27.5	<0.001		1
	Time × Group	1362.646	2	681.323	17.42	<0.001	0.21	1
	Error (Time)	9541.096	244	39.11				
Help-seeking Behavior	Time	49.876	1.85	26.95	2.44	0.094		0.469
	Time × Group	86.992	1.85	47.013	4.26	0.018	0.05	0.716
	Error (Time)	2492.825	225	11.043				

**Table 4:** Means and standard deviations of the scores of variables from pre-test to follow-up in intervention and control groups.

	Intervention Group (*n* = 62)			Control Group (*n* = 62)		
	Pre-Test	Post-Test	Follow-Up	Pre-Test	Post-Test	Follow-Up
Difficulties in Emotion regulation	32.56 (9.18)	27.35 (8.04)	27 (9.34)	31.21 (10.92)	30.31 (11.82)	29.03 (10.45)
Depressive symptoms	15.14 (8.31)	10.82 (7.64)	10.89 (9.33)	12.23 (9.54)	12.7 (10.34)	12.22 (10.26)
Mental well-being	47.73 (7.65)	51.73 (8.42)	52.19 (9.52)	52.11 (9.01)	50.9 (9.21)	51.05 (9.46)
Internalizing and externalizing problems	27.68 (14.33)	19.11 (13.26)	18.1 (12.9)	22.05 (14.96)	21.68 (15.8)	20.52 (15.32)
Help-seeking behavior	29.52 (6.31)	30.58 (6.44)	31.21 (6.36)	31.87 (3.74)	32.56 (4.9)	31.36 (4.94)

**Table 5:** Pairwise comparisons of pre- post-, and follow-up measurements of variables in the intervention group.

	Time (I)	Time (J)	Mean Differences (I-J)	SE	*p*	Lower Bound	Upper Bound
Difficulties in Emotion Regulation	1	2	5.21	0.69	<0.001	3.50	6.92
		3	5.56	1.01	<0.001	3.07	8.06
	2	1	−5.21	0.69	<0.001	−6.92	−3.50
		3	0.35	0.90	1.000	−1.87	2.58
	3	1	−5.56	1.01	<0.001	−8.06	−3.07
		2	−0.35	0.90	1.000	−2.58	1.87
Depressive Symptoms	1	2	4.32	0.64	<0.001	2.75	5.89
		3	4.25	0.89	<0.001	2.07	6.43
	2	1	−4.32	0.64	<0.001	−5.89	−2.75
		3	−0.07	0.74	1.000	−1.89	1.74
	3	1	−4.25	0.89	<0.001	−6.43	−2.07
		2	0.07	0.74	1.000	−1.74	1.89
Mental Well-being	1	2	−4	0.81	<0.001	−6.00	−2.00
		3	−4.47	0.93	<0.001	−6.76	−2.17
	2	1	4	0.81	<0.001	2.00	6.00
		3	−0.47	0.88	1.000	−2.62	1.69
	3	1	4.47	0.93	<0.001	2.17	6.76
		2	0.47	0.88	1.000	−1.69	2.62
Internalizing and Externalizing Problems	1	2	8.56	1.38	<0.001	5.18	11.95
		3	9.58	1.39	<0.001	6.15	13.01
	2	1	−8.56	1.38	<0.001	−11.95	−5.18
		3	1.02	1.21	1.000	−1.96	3.99
	3	1	−9.58	1.39	<0.001	−13.01	−6.15
		2	−1.02	1.21	1.000	−3.99	1.96
Help-seeking Behavior	1	2	−1.06	0.53	0.150	−2.37	0.25
		3	−1.7	0.63	0.026	−3.24	−0.16
	2	1	1.06	0.53	0.150	−0.25	2.37
		3	−0.63	0.66	1.000	−2.26	0.99
	3	1	1.7	0.63	0.026	0.16	3.24
		2	0.635	0.66	1.000	−0.99	2.26

**Table 6:** Pairwise comparisons of pre- post-, and follow-up measurements of secondary outcomes in the control group.

	Time (I)	Time (J)	Mean Differences (I-J)	SE	*p*	Lower Bound	Upper Bound
Difficulties in Emotion Regulation	1	2	0.9	0.81	0.805	−1.09	2.89
		3	2.18	0.76	0.017	0.31	4.04
	2	1	−0.9	0.81	0.805	−2.89	1.09
		3	1.27	0.83	0.388	−0.77	3.31
	3	1	−2.18	0.76	0.017	−4.04	−0.31
		2	−1.27	0.83	0.388	−3.31	0.77

**Table 7:** Comparison of changes over timepoints between intervention and control group.

	Intervention- Control	Mean Difference	SE	*p*	Lower Bound	Upper Bound
Difficulties in emotion regulation	Pre/Post	−4.31	1.07	<0.001	−6.42	−2.20
	Post/Follow-up	0.92	1.23	0.455	−1.50	3.35
	Pre/Follow-up	3.39	1.27	0.009	0.88	5.89
Depressive symptoms	Pre/Post	−4.79	0.98	<0.001	−6.72	−2.86
	Post/Follow-up	0.55	0.96	0.563	−1.34	2.45
	Pre/Follow-up	4.23	1.08	<0.001	2.08	6.38
Mental well-being	Pre/Post	5.21	1.02	<0.001	3.19	7.24
	Post/Follow-up	0.32	1.18	0.785	−2.01	2.66
	Pre/Follow-up	−5.53	1.12	<0.001	−7.76	−3.30
Internalizing/ Externalizing problems	Pre/Post	−8.19	1.68	<0.001	−11.52	−4.87
	Post/Follow-up	0.15	1.50	0.922	−2.83	3.12
	Pre/Follow-up	8.04	1.58	<0.001	4.91	11.18
Help-seeking behavior	Pre/Post	0.37	0.69	0.591	−0.99	1.73
	Post/Follow-up	1.84	0.86	0.034	0.15	3.54
	Pre/Follow-up	−2.21	0.88	0.013	−3.95	−0.47

## Data Availability

The data presented in this study are available on request from the corresponding author.
